# Full-Length Genome Sequence of a Variant Porcine Epidemic Diarrhea Virus Strain, CH/GDZQ/2014, Responsible for a Severe Outbreak of Diarrhea in Piglets in Guangdong, China, 2014

**DOI:** 10.1128/genomeA.01239-14

**Published:** 2014-12-04

**Authors:** Deping Song, Yanjun Chen, Qi Peng, Dongyan Huang, Tiansheng Zhang, Tao Huang, Fanfan Zhang, Xinrong Zhou, Yuxin Tang

**Affiliations:** Department of Preventive Veterinary Medicine, College of Animal Science and Technology, Jiangxi Agricultural University, Nanchang, Jiangxi, China

## Abstract

The full-length genome sequence of a variant porcine epidemic diarrhea virus (PEDV) strain, CH/GDZQ/2014, was determined. The isolate was a variant strain with a relatively far relationship with the PEDV strains previously identified in the same area between 2011 and 2012 and was genetically distinct from the CV777-based vaccine strain currently being used in China.

## GENOME ANNOUNCEMENT

Porcine epidemic diarrhea virus (PEDV), an enveloped, single-stranded RNA virus, is a member of the genus *Alphacoronavirus* (1, 2). Field strains of PEDV, which are genetically distinct from the CV777-based vaccine strain, have caused severe epidemics of diarrhea in swine herds in China since 2010. The mortality rate in neonatal piglets affected with variant field strains of PEDV can reach as high as 100% (3–7). Here, we report the full-length genome sequence of a variant PEDV isolate, CH/GDZQ/2014, which is responsible for a severe outbreak of diarrhea in Guangdong, southern China, in March 2014.

Fecal samples were collected from piglets infected with PEDV. Afterward, viral RNA was extracted, and then the full-length genome sequence of CH/GDZQ/2014 was amplified by 33 pairs of primers specific to PEDV. The amplicons obtained were cloned into the PMD18-T vector (TaKaRa, Japan) and sequenced. The complete genome sequence was assembled and annotated by using DNAStar Lasergene version 7.10 (DNAStar, Inc., Madison, WI). Nucleotide and amino acid sequences along with 32 reference PEDV sequences retrieved from GenBank were comparatively analyzed.

The complete genome sequence of CH/GDZQ/2014 is 28,038 nucleotides (nt) in length, excluding the 3′ poly(A) tail. The genomic organization of the virus is arranged in the order of 5′ untranslated region (UTR)-replicase (1a/1b)-spike (S)-open reading frame 3 (ORF3)-envelope (E)-membrane (M)-nucleocapsid (N)-3′ UTR. The 5′ UTR of CH/GDZQ/2014 is 292 nt in length; the replicase, consisting of 1a and 1b is 20,345 nt in length; and the S, ORF3, E, M, and N genes and 3′ UTR are 4,161 nt, 675 nt, 231 nt, 681 nt, 1,326 nt, and 334 nt in size, respectively.

Phylogenetic analysis demonstrated that the CH/GDZQ/2014 strain had a relatively far relationship with four variant PEDV strains (CH/GD-01, CH/GDGZ/2012, GD-A, and GD-1) identified in Guangdong between 2011 and 2012 (8–11), indicating that CH/GDZQ/2014 was genetically distinct from these strains. Sequence homology analysis showed that CH/GDZQ/2014 had 96.7% nt identity with the prototype CV777 strain (12) but shared 97.8 to 99.0% nt identity with the variant PEDV strains that have emerged since 2010 in China (13, 14) and 98.8% nt identity with two newly identified U.S. strains (15, 16). The S protein of CH/GDZQ/2014 was 4,161 nt long, encoding a protein of 1,386 amino acids, and had 93.1 to 98.6% nt identity with the reference strains, showing the lowest identity with the CV777-based vaccine strain (NC_003436) (17) and the highest identity with two Chinese strains (BJ-2011-1 and JS-HZ/2012). Compared to the deduced amino acid (aa) sequences of the S gene of CV777, CH/GDZQ/2014 showed 5 aa insertions (59QGVN62, 139N140), 2 aa deletions (161GK162), and 94 aa mutations. Of these variations, most were located in the N-terminal of the S protein (18, 19).

CH/GDZQ/2014 was a very virulent field PEDV strain isolated from Guangdong Province in southern China. The genome data provided here will help better understand molecular characteristics of variant field strains of PEDV currently affecting swine in Guangdong Province.

### Nucleotide sequence accession number.

The complete genome sequence of PEDV strain CH/GDZQ/2014 was deposited in GenBank under the accession number KM242131.
